# Grafting of Lactic Acid and ε-Caprolactone onto Alpha-Cellulose and Sugarcane Bagasse Cellulose: Evaluation of Mechanical Properties in Polylactic Acid Composites

**DOI:** 10.3390/polym16212964

**Published:** 2024-10-23

**Authors:** Oscar Salvador Valle Reyes, Eulogio Orozco-Guareño, Rosaura Hernández-Montelongo, Abraham Gabriel Alvarado Mendoza, Liliana Martínez Chávez, Rubén González Núñez, Jacobo Aguilar Martínez, Francisco Javier Moscoso Sánchez

**Affiliations:** 1 Departamento de Química, Centro Universitario de Ciencias Exactas e Ingenierías, Universidad de Guadalajara, Guadalajara 44430, Mexico; oscar.valle@alumnos.udg.mx (O.S.V.R.); eulogio.orozco@academicos.udg.mx (E.O.-G.); gabriel.alvarado@academicos.udg.mx (A.G.A.M.); 2 Departamento de Bioingeniería Traslacional, Centro Universitario de Ciencias Exactas e Ingenierías, Universidad de Guadalajara, Guadalajara 44430, Mexico; 3 Departamento de Farmacología, Universidad de Guadalajara, Blvd. Marcelino García Barragán No. 1421, Guadalajara 44430, Mexico; liliana.mchavez@academicos.udg.mx; 4 Departamento de Ingeniería Química, Centro Universitario de Ciencias Exactas e Ingenierías, Universidad de Guadalajara, Guadalajara 44430, Mexico; ruben.gnunez@academicos.udg.mx; 5 Departamento de Ciencias Tecnológicas, Centro Universitario de la Ciénega, Universidad de Guadalajara, Av. Universidad 1115, Ocotlán 47820, Mexico; jacobo.aguilar@academicos.udg.mx

**Keywords:** poly acid lactic, lactic acid, ε-caprolactone, alpha-cellulose, sugarcane bagasse cellulose, surface modification, grafting polymerization

## Abstract

In this paper, we present the synthesis of composite materials comprised of α-cellulose and sugarcane bagasse cellulose fibers grafted with lactic acid and ε-caprolactone. These fibers were incorporated as reinforcements into a PLA matrix by extrusion, producing composite materials with improved mechanical properties. The grafting of lactic acid and ε-caprolactone onto the fibers was confirmed by FTIR spectroscopy, demonstrating the chemical modification of the fibers. The morphology of the fibers and composites was analyzed through scanning electron microscopy (SEM), showing that the fibers are encapsulated within the polymeric matrix. This suggests good PLA–fiber interaction for the 90 PLA/10 α-Cel, 90 PLA/10 LAC-g-α-Cel, and 90 PLA/10 ε-CL-g-α-Cel composite materials. The obtained composite materials were tested under tensile loading. Incorporating 10 wt% of LAC-g-FBA-Cel and α-Cel-g-FBA-Cel grafted fibers into the PLA matrix improved the tensile modulus by 28% and 12%, respectively, compared with PLA. The maximum tensile strength values obtained were for composite materials with 10 wt% PLA/α-Cel, LAC-g-α-Cel, and FBA-Cel with 23, 27, and 37% concerning PLA. DSC thermal studies showed a reduction in the glass transition temperature in the composites with grafted fibers. The results suggest better interfacial adhesion between the PLA matrix and both grafted and non-grafted α-cellulose fibers, which contributes to the observed improvements in the mechanical and thermal properties of the composite materials. The results demonstrate that the composites can be produced through extrusion. Once the optimal concentration has been determined, α-cellulose or sugarcane bagasse grafted with lactic acid and ε-caprolactone can be incorporated into the PLA matrix, exhibiting adjustable properties.

## 1. Introduction

Nowadays, environmental concerns have driven the search for more sustainable materials. Growing concern about the environmental impact of petroleum-based plastics has driven research into biodegradable materials. Among these is polylactic acid (PLA), a renewable polymer that has gained popularity due to its biodegradability and ability to be used in packaging applications, biomaterials, and more [1]. On the other hand, cellulose, the most abundant organic polymer on Earth, has emerged as an attractive alternative for polymer reinforcement due to its low density, biodegradability, and excellent mechanical properties. However, its processing presents challenges due to its hydrophobic nature, which makes it difficult to disperse the fibers and adhere them to polymeric matrices. A promising solution is the surface modification of cellulose to improve its compatibility. This modification, which involves substituting hydroxyl groups through chemical reactions, allows the mechanical properties and biodegradability of the cellulose to be preserved. Grafting polymers onto cellulose effectively enhances physical and chemical interactions in polymeric matrices [2]. Developing new reaction pathways to modify cellulosic fibers using biodegradable monomers such as lactic acid (LAC) and ε-caprolactone (ε-CL) offers a viable alternative for fabricating advanced biomaterials. The main difficulty in graft polymerization lies in the presence of water in cellulose [3]. This is because most catalysts used for ε-CL or LAC copolymerization are moisture-sensitive. However, polylactic acid (PLA) products exhibit several drawbacks, such as low toughness, moisture sensitivity, a low oxygen barrier, poor hydrophilic behavior, and a low degradation rate, which limits their commercial applications [4]. A valuable strategy to improve these properties without compromising biodegradability is blending PLA with other biopolymers. This improvement can be achieved by adding various types of grafted cellulosic materials.

For example, in 2002, Teramoto et al. (2002) [5] studied the effect of temperature, reaction time, and amount of monomer feed on the progress of the graft copolymerization reaction of ε-CL and LAC on cellulose diacetate (CDA) at the hydroxyl group positions. They obtained a thermoplastic material with good tensile strength. Lönnberg et al. (2006) [6] grafted onto a cellulose paper surface poly(ε-caprolactone) (PCL) and poly(L-lactic acid) (PLLA), showing that the grafting became more efficient when the pretreatment was performed with 2,2-bis(methylol)propionic acid and that an appropriate ratio of initiator can control the amount of grafted polymer. Lönnberg et al. in 2011 [7] studied the grafting of ε-CL polymers using ring-opening polymerization of ε-CL and obtained cellulose grafted with poly(ε-caprolactone) (PCL). The variation in the PCL graft length allowed the material’s toughness to be modified. Muiruri et al. [8] synthesized nanocrystals with rubber and a graft of poly(L-lactide). The formation of stereo complexes improved the thermal properties of the nanocomposites, improving the melting temperature and breaking stress.

**Figure 1:**
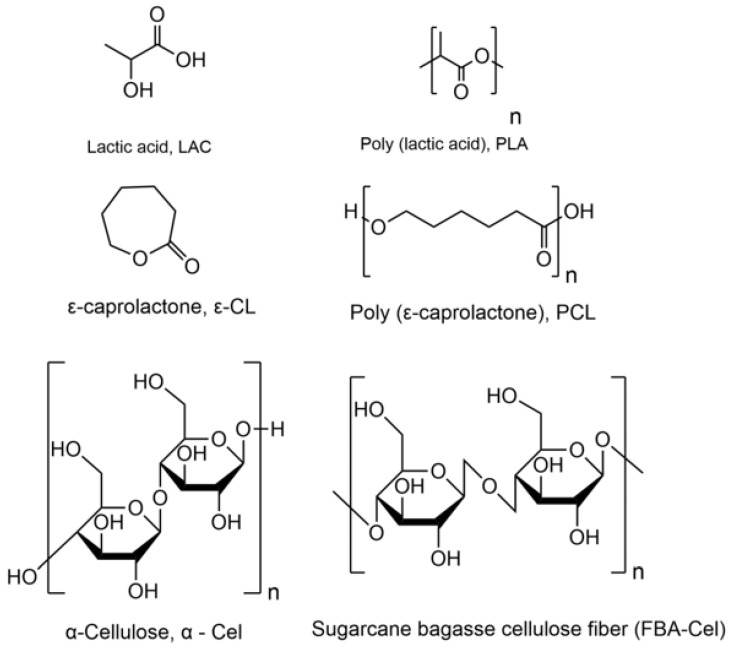
Chemical structure of LAC, PLA, ε-CL, PCL, α-Cel, and FBA-Cel.

Gupta and Katiyar (2017) [9] grafted poly(D-lactic acid) (PDLA) onto cellulose microcrystals (CMCs). They formed complex stereo crystallites in the poly(L-lactic acid) (PLLA) matrix. The tensile strength and permeability of oxygen and water vapor were improved compared with the original PLLA–PDLA blend. Yu et al. 2018 [10] synthesized microcrystalline cellulose-grafted PCL (MCC-g-PCL) by 4-dimethylamino pyridine-catalyzed ring-opening copolymerization in a solvent system of tetrabutylammonium acetate and dimethyl sulfoxide, blending it with graphene (GO) to form GO–MCC-g-PCL hybrid films. The composite exhibited better mechanical properties versus the MCC-g-PCL, which were attributed to the strong hydrogen cross-linking interaction of GO and MCC-g-PCL. As can be seen, cellulose presents compatibility challenges when combined with hydrophobic polymers such as PLA due to the difference in their surface characteristics. This study focused on fabricating and characterizing composite materials formed with cellulose fibers and sugarcane bagasse cellulose modified with LAC and ε-CL and their incorporation into a PLA matrix. The main objective was to evaluate the impact of LAC and ε-CL grafting on the mechanical, thermal, and morphological properties of the PLA-grafted cellulose material composites. Figure 1 shows the chemical structure of all raw materials employed in this study.

## 2. Materials and Methods

### 2.1. Materials

The alpha-cellulose (α-Cel) CAS No. 9004-34-6 with a bulk density of 5.6–6.8 (CC/g) and a particle size of 35 Mesh < 20.0, (% Retained) 200 Mesh > 35.0, and (% Passing) 100 Mesh > 50.0 from Aldrich (Sigma Aldrich reagent grade, St. Louis, MO, USA) was used. Sugarcane (Saccharum officinarum) bagasse cellulose fiber (FBA-Cel) was obtained from the local market. It was ground using a PULVEX blade mill mark (Cd. Mexico, Mexico) and sieved through an RO-TAP RX29 (Cleveland, OH, USA). The material of this study was retained in a mesh of 850 to 106 μm in a series of Tyler sieves (Mentor, OH, USA). The FBA-Cel extracted from the sugarcane fiber was obtained using a method developed at the Scientific Research Center of Yucatán (CICY), Mexico [11]. The cellulose chemical content was 72.4% holocellulose, 20.07% lignin, 5.4% removable, and 1.32% ash. For the synthesis, lactic acid (LAC) CAS No. 50-21-5, tin octoate (Sn(Oct)_2_) CAS No. 301-10-0, ε-caprolactone (ε-CL) CAS No. 502-44-3, benzyl alcohol CAS No. 100-51-6, toluene CAS No. 108-88-3, methanol (MeOH) CAS No. 67-56-1, ethyl acetate CAS No. 141-58-6, tetrahydrofuran (THF) CAS No. 1693-74-9, sulfuric acid (H_2_SO_4_) CAS No. 7664-93-9, sodium hydroxide (NaOH) CAS No. 1310-73-2, acetone CAS No 67-34-1, 1-carbonyldiimidazole, dimethylsulfoxide CAS No. 67-68-5, and sodium hypochlorite (NaClO) CAS No. 7681-52-9 were used without further purification. All analytical grade reagents were obtained from Sigma Aldrich (St. Louis, MO, USA), and distilled water was purchased from Selectropura Products (Guadalajara, Mexico). The PLA resin from Nature Works Ingeo Biopolymer 3251D, with a melting temperature range of 188–210 °C, a melt index of 35 g/10 min (190 °C, 2.16 kg), and a density of 1.24 g/cm^3^, was used (Minnetonka, MN, USA).

### 2.2. Surface Modification of α-Cel or FBA-Cel by Lactic Acid

In the first method, 30 g of α-Cel or FBA-Cel was mixed in 200 mL of tetrahydrofuran (THF) in a 500 mL reactor. Subsequently, 20 mL of LAC was added dropwise at 40 °C and maintained for 30 min. At the end of the reaction time, the sample was removed from the reaction system and placed in a vacuum rotary evaporator to evaporate the THF and volatiles. Subsequently, 30 g of α-Cel or FBA-Cel was placed in a 500 mL flask and mixed with 200 mL of toluene. The mixture was heated to 100 °C in a thermo-oil bath and maintained for 10 h. The α-Cel or FBA-Cel sample, now treated with LAC, was then removed and transferred to a vacuum filter, where it was washed five times with excess THF and ethyl acetate to eliminate any remnant unreacted LAC. The sample was dried in an oven at 50 °C until a constant weight was achieved [2,12]. 

### 2.3. Surface Grafting of α-Cel and FBA-Cel Modified via In Situ Polymerization

In this procedure, in a 500 mL flask, α-Cel or FBA-Cel with LAC was added in a ratio of 1:2 by weight. Then, 0.05% by weight of Sn(Oct)_2_ was added as a catalyst, and 100 mL of toluene was added for every 10 g of α-Cel or FBA-Cel. The flask containing the α-Cel or FBA-Cel sample and the solution was placed on a thermal plate with constant stirring and heated to 100 °C for 48 h [2,12]. After this period, the α-Cel or FBA-Cel modified with LAC was transferred to a vacuum filter and washed five times with excess ethanol to remove any remnant unreacted LAC and catalyst residues. The sample was then placed in an oven at 50 °C until a constant weight was achieved. The product obtained from the above-described method was the grafted copolymer comprised of PLA and α-Cel (LAC-g-α-Cel) or PLA and sugarcane bagasse cellulose fiber (LAC-g-FBA-Cel).

### 2.4. The Polymerization Procedure of ε-CL Initiated with Sn(Oct)_2_ for α-Cel or FBA-Cel

In a 250 mL three-necked flask, 15 mL of the monomer of ε-CL and 0.7 mL of the co-initiator benzyl alcohol were added. Subsequently, the reactor was then sealed with a rubber stopper, and the reaction mixture was kept under a stirrer for two hours. Afterward, the flask and the solution were immersed in an oil bath with stirring, and 1% by weight of Sn(Oct)_2_ (catalyst) was added concerning the total amount of ε-CL added. The mixture was heated to 140 °C, and the polymerization was carried out for 5 h. The product was dispersed in toluene to −6 °C, precipitating the polymer, and the monomer and non-grafted prepolymer were removed from the mixture after washing the product with excess methanol. The final product was introduced into an oven at 50 °C to evaporate the solvent until a constant weight of the polymer was achieved [13]. Subsequently, in a 500 mL Schlenk flask, 15 g of α-Cel or FBA-Cel and a prepolymer of ε-CL were added in a 1:1 ratio. After 1% by weight of 1-carbonyldiimidazole in 200 mL of dimethylsulfoxide was added into the reactor, the reaction was carried out for 16 h at room temperature with constant stirring [14]. The product was vacuum-filtered and washed with methanol, followed by acetone, to remove ungrafted polymer and solvent residues. All solvents and residual materials were responsibly collected and processed by an external company to ensure proper environmental protection and prevent contamination.

### 2.5. Manufacture of the Composite Material of PLA and Modified or Unmodified α-Cel and FBA-Cel

The composite material was manufactured using a twin-screw extruder from Thermo Scientific (Karlsruhe, Germany). The modified fibers added to the PLA matrix were 5% and 10% (*w*/*w*). These were fed directly into the feed hopper, the temperature profile in the screw was 170,170, 180, 180, 185, 185, and 190 °C, the exit die had a 2 mm hole, and the screw speed was 200 rpm. The composite material was dried in an oven at 50 °C for three days and then pelletized. A determined quantity of pellets was deposited into a 12 × 12 × 0.3 cm metal mold. This was introduced into a Carver Inc. brand thermo press (Wabash, IN, USA) at a temperature of 170 °C without pressure for 4 min. Then, the pressure was increased to 100 kg/cm^2^ and maintained for 4 min. At the end of this period, the heat of the thermo press was disconnected, and the pressure was maintained, allowing the system to cool by convection to room temperature. Later, the plate was removed.

### 2.6. Characterization

#### 2.6.1. Scanning Electron Microscopy (SEM)

Composite material samples were placed in liquid nitrogen and subsequently fractured. The broken samples were coated with a thin conductive layer of Au under vacuum using an SPI Module Sputter Coater for 20 s. The fibers and composites were analyzed in a Hitachi TM-1000 field-emission scanning electron microscope (Hitachi, Tokyo, Japan). 

#### 2.6.2. Fourier Transform Infrared (FTIR) Spectroscopy by Attenuated Total Reflectance (ATR)

The FTIR analysis of the modified fibers and composite material was performed by a Thermo Scientific iS5 Nicolet (Thermo Fisher Scientific, Madison, WI, USA) with ATR. The spectra were analyzed at a 4 cm^−1^ resolution with 24 scans in a range from 400 cm^−1^ to 4000 cm^−1^. All the samples were oven-dried at 60 °C for 24 h before testing.

#### 2.6.3. Mechanical Properties

An Instron universal testing machine (Model 3345, Norwood, MA, USA) with a 1 kN load cell was used to determine the mechanical properties. The specimens were cut using a Guian laser (model gn640ms, Jinan, China). The tensile test was carried out according to ASTM D638 [15]. The gauge length was set at 25.4 mm, and the cross-head testing speed was 1 mm/min at 25 °C. Five samples were used to report the average and standard deviation. The tensile strength and Young’s modulus were analyzed.

The flexural test samples were analyzed following the procedure of the ASTM D790 [16], using a three-point contact system, in the universal testing machine at 1 kN at a speed of 1 mm/min. Five samples were used to report the average and standard deviation of the flexural test. All tests were recorded and the results automatically calculated by the Instrum Bluehill 3 software.

The following nomenclature is used to describe the samples. Alpha-cellulose is abbreviated as α-Cel, and sugarcane bagasse cellulose fiber is abbreviated as FBA-Cel. The modification of alpha-cellulose and bagasse cellulose fiber grafted with LAC or ε-CL is abbreviated as LAC-g-α-Cel and ε-CL-g-FBA-Cel, respectively. Composites of % PLA and % α-Cel modified with a PLA graft are abbreviated as % PLA/% PLA/LAC-g-α-Cel, and composites of PLA and % FBA-Cel modified with a PLA graft are abbreviated as % PLA/% LAC-g-FBA-Cel, where % is percentage weight.

#### 2.6.4. Differential Scanning Calorimetry (DSC)

Thermal analysis was realized with a Discovery TA (model: Q100, TA Instruments, New Castle, CA, USA) analyzer. The samples were dried in an oven at 50 °C for 24 h. In the first scan, 5–10 mg samples were heated from 20 °C to 200 °C and kept at 200 °C for 1 min to eliminate small volatile molecules. In the second scan, the samples were cooled to 20 °C, kept for 1 min, subsequently heated to 200 °C, kept for 1 min, and then finally cooled to 20 °C. All experiments had a heating rate of 10 °C/min and a 50 mL/min flow of nitrogen gas. The T_g_ and degree of crystallinity (*X_c_*) of the PLA and its composite material were calculated with the second scan. The degree of crystallinity was calculated using the equation

(1) $$X_{c}=\left(\frac{∆H_{m}-∆H_{c}}{∆H_{mo}\times w}\right)$$

where Δ*H_m_* is the melting temperature, Δ*H_mo_* is the melting enthalpy of a crystalline PLA material (taken as 93 J/g) [17], Δ*H_c_* is the cold crystallization enthalpy, and w is the mass fraction of PLA in the composite material. 

#### 2.6.5. X-Ray Photoelectron Spectroscopy (XPS) Measurements

The photoelectron spectra were analyzed on a SPECS Phoibos 150 spectrometer (Berlin, Germany) equipped with an Al X-ray source Kα (1486.7 eV). The sample was irradiated in a vacuum chamber (pressure below 10^−9^ Torr). The spectral analysis (curve fitting) was carried out using the XPSPEAK4.1 (Department of Chemistry, The Chinese University of Hong Kong, Shatin, Hong Kong) software. The binding energies were fixed at 284.8 eV and shifted according to the carbon–carbon bonding.

## 3. Results and Discussion

### 3.1. Reaction Scheme and Morphology of Grafted Fibers

**Figure 2:**
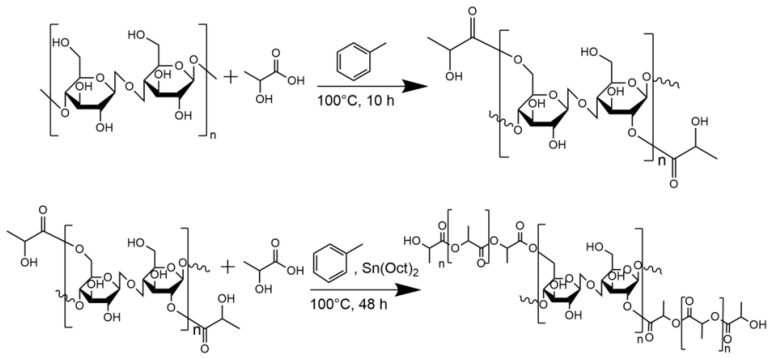
Reaction scheme for the formation of LAC-g-α-CEL or LAC-FBA-Cel.

The surface modification reaction of α-Cel or FBA-Cel with LAC is presented in Figure 2. First, α-Cel or FBA-Cel is azeotropically dehydrated with toluene. Then, hydroxyl groups on the surface of α-Cel or FBA-Cel react with LAC to form carboxylate bonds of α-Cel or FBA-Cel. In the presence of Sn(Oct)_2_, lactide and hydroxyl groups on the surface of α-Cel or FBA-Cel coordinate with Sn(Oct)_2_, leading to the cleavage of the acyl-oxygen bond of L-lactide and the activation of the hydroxyl group [3]. The polymerization is based on the fixation of Sn–O functionalities on the surface of α-Cel by the in situ reaction of Sn(Oct)_2_ with the hydroxyl groups of modified α-Cel or FBA-Cel. The first is mainly considered a graft copolymer (LAC-g-α-CEL or LAC-FBA-Cel), and the second is an extension of the homopolymer. The percentage grafting (*P_g_*) was calculated according to Equation (2), following the procedure previously reported [18,19]. The *P*_g_ for LAC-g-α-Cel was determined at 60% and for LAC-g-FBA-Cel it was determined at 56%. The values given are the average of all copolymerization reactions.

(2) $$P_{g}=\frac{W_{g}-W}{W}\times 100$$

where *W_g_* represents the mass of the fibers after the grafting reaction and *W* represents the mass of the fibers before the copolymerization reaction.

**Figure 3:**
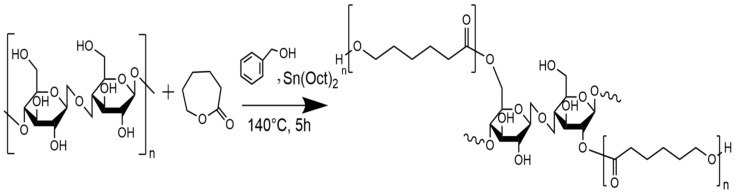
Reaction scheme for the formation of ε-CL-g-FBA-Cel.

Surface modification of α-Cel or FBA-Cel fibers by ε-CL grafting was performed via ring-opening polymerization (ROP) of ε-CL as shown in Figure 3. This heterogeneous process uses Sn(Oct)_2_ as a catalyst and benzyl alcohol as a solvent. Sn(Oct)_2_ activates the ε-CL monomer, facilitating ring opening and the formation of PCL chains. Hydroxyl groups on the fiber surface act as initiating sites for the growth of PCL chains, which are grafted directly onto the fibers, forming a PCL layer. The reaction is stopped by cooling. The *P*_g_ was 58% for LAC-g-FBA-Cel and 53% for ε-CL-g-FBA-Cel.

**Figure 4:**
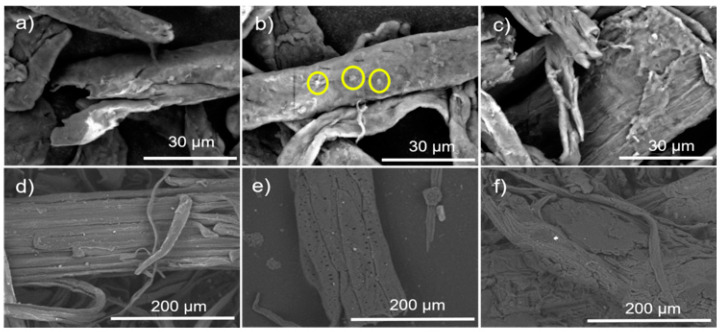
Micrographs of: (**a**) α-Cel, (**b**) LAC-g-α-Cel, (**c**) ε-CL-g-α-Cel, (**d**) FBA-Cel, (**e**) LAC-g-FBA-Cel), and (**f**) ε-CL-g-FBA-Cel.

Figure 4a–c shows the SEM micrographs of the morphology of the α-Cel with or without treatment. Figure 4a shows the dispersion of the α-Cel fibers in single fibers. The fibers present different diameters and lengths, and some cellulose fibers are broken. Figure 4b shows a fiber surface with small voids (yellow circles) caused by the reaction of the fiber with lactic acid and small clusters that were deduced to be polymerized PLA that is not distributed homogeneously on the fiber surface. Figure 4c shows a somewhat roughened cellulose fiber surface portion, with a layer partially covered by ε-CL, displaying a subtle shine that is slightly more reflective than pure cellulose fiber. Figure 4e–f displays micrographs of FBA-Cel without or with chemical modification. Figure 4d presents the structure of FBA-Cel naturally with rigid and ordered fibrils [20]. However, after treatment with LAC, the appearance of holes on the surface (Figure 4e) suggests that the LAC and the reaction conditions may have modified the surface of FBA-Cel [21]. The average values of the diameters of the cellulose fibers are α-Cel = 19.54 ± 3.1, LAC-α-Cel = 60.73 ± 9.5, ε-CL-α-Cel = 28.0 ± 3.7, FAB-Cel = 78.94 ± 11.4, LAC-FAB-Cel = 79.7 ± 9.3, and ε-CL-FAB-Cel = 88.42 ± 12.5. All values are in microns.

### 3.2. Structural Changes Evaluated by FTIR

**Figure 5:**
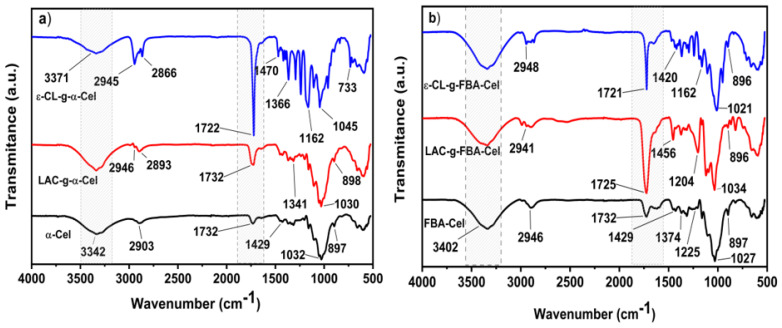
FTIR spectra of (**a**) α-Cel modified with PLA and PCL; and (**b**) FBA-Cel modified with PLA and PCL.

The FTIR spectra of α-Cel, FBA-Cel, and the chemical modification of fibers are shown in Figure 5. α-Cel and FBA-Cel display similar bands at 3342–2903 cm^−1^, the stretching vibration of OH and C–H bonds in polysaccharides. Typical cellulose bands in the region of 1732–900 cm^−1^ were observed. The absorption bands of 1429 to 1032 cm^−1^ and 897 cm^−1^ belong to stretching and bending vibrations of -CH_2_, -CH, -OH, and C–O bonds in cellulose. Bands to 1429 cm^−1^ and 897 cm^−1^ are associated with cellulose’s crystalline structure and the amorphous region [22]. Al alterations were observed in the FTIR spectra after grafting LAC onto the surface of α-Cel (LAC-g-α-Cel) via esterification. The carbonyl (C=O) band, characteristic of ester groups, emerges at approximately 1732 cm⁻^1^, indicating a shift from the original LAC carbonyl band at 1750 cm⁻^1^ [23]. Additionally, there is a reduction in the intensity of the hydroxyl (OH) band of cellulose around 3342 cm⁻^1^, attributed to the formation of ester bonds. These spectral changes provide evidence of the grafting of LAC onto the cellulose surface through ester linkages. The functional groups involved in the interaction of ε-CL with cellulose include hydroxyl and ester groups. Absorption bands of cellulose or polyester hydroxyl groups are visible in the range of 3371 cm⁻^1^ and the C–H vibration in the range of 2945 cm^−1^. When PCL is grafted onto cellulose, ester linkages are formed between the hydroxyl groups of cellulose and the carboxyl groups of ε-CL. FTIR spectra of the grafts show absorptions around 1722 cm^−1^, which is indicative of the C=O vibration of the ester group. Absorptions characteristic of aromatic groups were detected at 700 and 733 cm^−1^. This is a consequence of C–H vibrations due to the substitution of benzylic groups at the position of the cellulose backbone. Bands at 1446, 1371, and 1341 cm^−1^ are bending vibrations of ―CH_3_ and ―CH, the band at 1268 cm^−1^ belongs to stretching vibrations of ―C―O―C―, bands at 1162 and 1030 cm^−1^ are the symmetric and asymmetric stretching vibration ―C―O―C― and CH_3_ rocking, and the band at 898 cm^−1^ belongs to ―C―COO- [24]. On the other hand, the LAC-g-FBA-Cel and ε-CL-g-FBA-Cel showed similar characteristic bands in CH_2_, asymmetric and symmetric stretching at 2946 and 2866 cm^−1^, and the bands at 1721 and 1732 cm^−1^ are C=O stretching vibrations. The bands at 1374 and 1429 cm^−1^ are CH_2_ bending modes. Stretching vibrations at 1027, 1162, and 1225 cm⁻^1^ correspond to the band C—O—C. The bands at 1162 and 1293 cm^−1^ are assigned to C—O and C stretching in the amorphous and crystalline phases, respectively [25]. This allows for a comparison of the polymer-grafted cellulose substrates. In particular, in the band close to 1732 cm^−1^, the FTIR spectra of α-Cel and FBA-Cel showed a small adsorption band from the carbonyl group at circa 1732 cm^−1^. After that, α-Cel and FBA-Cel were grafted with LAC and ε-CL. The modified bands show a change in the adsorption intensity of the carbonyl group, confirming the presence of the grafted polymer. A similar result was obtained by Lönnberg et al. [6].

### 3.3. Composite Morphology Evaluated by SEM

**Figure 6:**
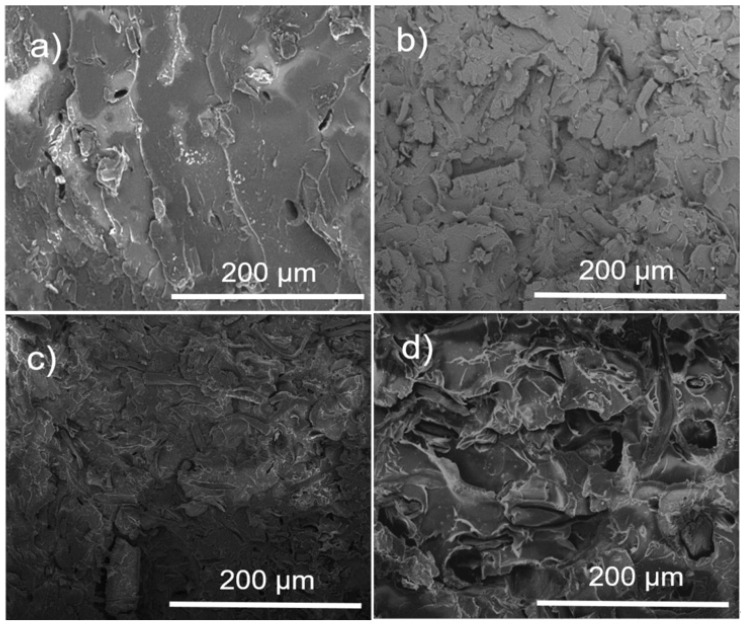
SEM micrographs of the PLA and composite materials at 500x magnification. (**a**) PLA, (**b**) 90 PLA/10 α-Cel, (**c**) 90 PLA/10 LAC-g-α-Cel, and (**d**) 90 PLA/10 ε-CL-g-α-Cel.

The morphology of the PLA and PLA–α-Cel fibers in the composite materials was investigated by SEM, as shown in Figure 6. Figure 6a shows the smooth surface of the PLA. Figure 6b–d shows the individual separation and dispersion of the cellulose fibers with or without a graft of LAC and ε-CL. This indicates that the grafted and ungrafted cellulose fibers were separated during the extrusion process and dispersed well within the PLA matrix. However, certain fibers exhibit strong adhesion to the matrix, while others show signs of breaking or tearing. Figure 4b,c shows that the fibers are encapsulated by a thin matrix layer, promoting better stress transfer between the matrix and reinforcing fibers [26]. By comparing Figure 6b,c with Figure 6d, it can be seen that there are fewer small voids between the fibers and the matrix in the 90 PLA/10 α-Cel and 90 PLA/10 LAC-g-α-Cel composites compared with the 90 PLA/10 ε-CL-g-α-Cel composite. In addition, the fiber detachments in Figure 6d are more evident than in Figure 6b,c. These voids within the matrix slightly reduce the tensile strength, although the impact is minimal.

**Figure 7:**
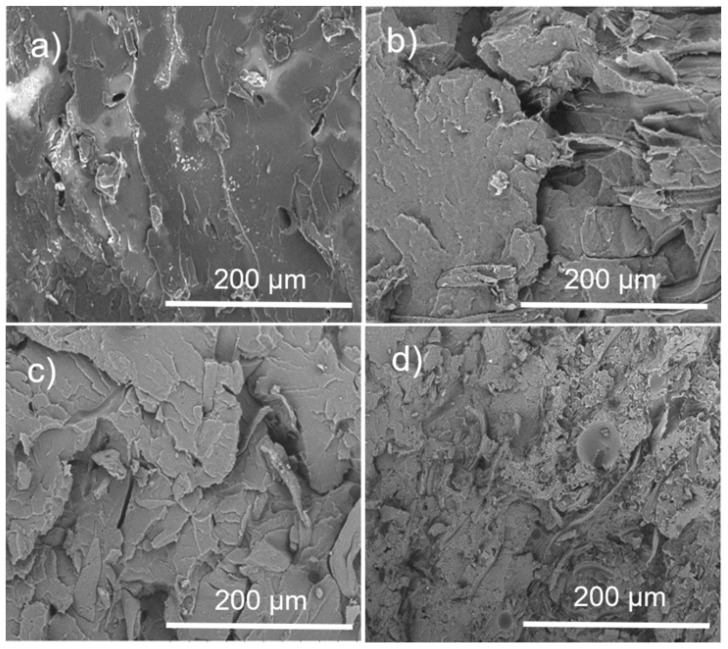
SEM micrographs of the PLA and composites materials at 500x magnification. (**a**) PLA, (**b**) 90 PLA/10 FBA, (**c**) 90 PLA/10 LAC-g-FBA, and (**d**) 90 PLA/10 ε-CL-g-FBA.

Figure 7 presents micrographs of PLA and PLA composite materials with grafted and ungrafted FBA-Cel. In Figure 7a, the micrograph of pure PLA shows a fracture characteristic of brittle materials with scattered small voids. In contrast, the micrographs in Figure 7b–d reveal a fracture of embedded fibers, with small interfacial voids between the PLA matrix and both grafted and ungrafted FBA-Cel, suggesting a weak interfacial bond. In addition, these micrographs show small, isolated cracks at the PLA–fiber interfaces [27]. This behavior indicates that the material does not experience a continuous catastrophic rupture; instead, the energy is distributed in different areas, slowing down the crack progression. This phenomenon suggests improving the impact resistance of the composites containing both grafted and ungrafted FBA-Cel. In the 90 PLA/10 ε-CL-g-FBA-Cel system shown in Figure 7d, microdroplets of ε-CL were observed to be dispersed throughout the PLA matrix. These microdroplets, which originate from low-molecular-weight ε-CL, can improve the toughness of the composite by functioning as local plasticizers within the matrix, thereby increasing the energy absorption capacity of the material.

### 3.4. PLA Composites with α-Cel and FBA-Cel

**Figure 8:**
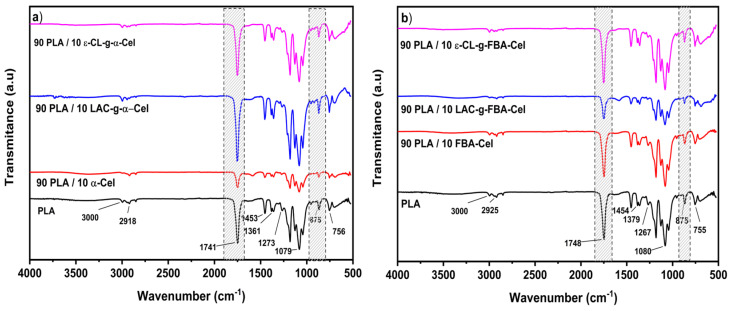
FTIR spectra of (**a**) PLA and the composite materials PLA/LAC-g-α-Cel and PLA/ε-CL-g-α-Cel and (**b**) PLA and the composite materials PLA/LAC-g-FBA and PLA/ε-CL-g-FBA.

Figure 8a,b shows the FTIR spectra of the composite materials and the PLA matrix. In general, the spectra present similar bands; there is only a difference in intensity. The spectra of PLA present two bands at 3000 cm^−1^ and 2918 cm^−1^ assigned to the asymmetrical and symmetrical -CH3 stretching. For the composite materials 90 PLA/10 α-Cel, 90 PLA/10 LAC-g-α-Cel, and 90 PLA/10 ε-CL-g-α-Cel, the asymmetrical and symmetrical -CH_3_ stretching is at 2998 cm^−1^ and 2850 cm^−1^. The bands at 1749–1751 cm^−1^ were attributed to the carboxylic stretching, the bands at 1361–1453 cm^−1^ represent the -CH deformation, the bands at 1079–1273 cm^−1^ correspond to the asymmetric C-O-C stretching, and those at 756 cm^−1^ represent the vibration of the -CH_2_. The band at 875 cm⁻^1^ of the composite material was attributed to the C–COO stretching vibration. This is commonly related to the ester linkages present in the structure of the polymers. The 875 cm⁻^1^ band could arise from interactions involving the crystalline structure of one of the components, possibly reflecting polymer chain conformations or ordering [28]. This is indicative of the interaction of the grafting fiber and PLA matrix.

**Figure 9:**
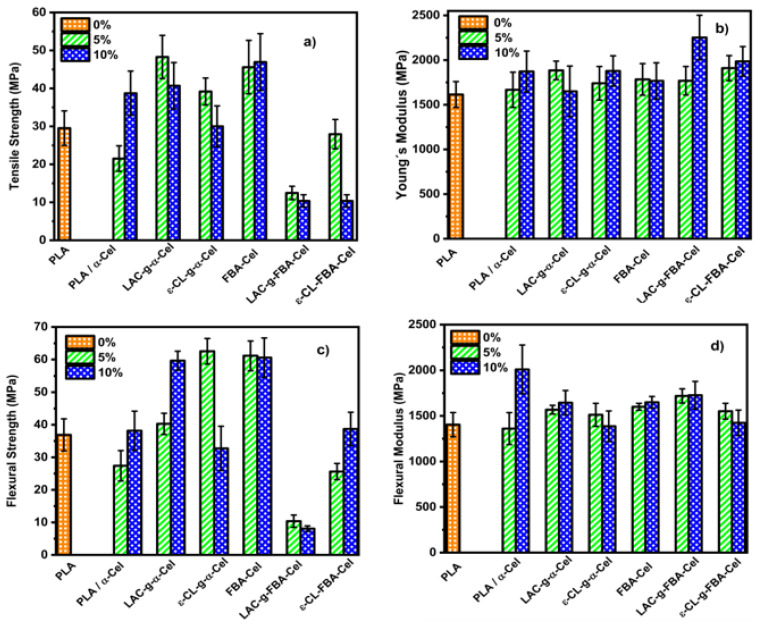
Mechanical properties of PLA and composites of PLA/grafting of fiber with different PLA and PCL contents. (**a**) Tensile strength, (**b**) Young’s modulus, (**c**) flexural strength, and (**d**) flexural modulus.

Figure 9 presents the mechanical properties of PLA and its composites with α-Cel and FBA-Cel, both grafted and ungrafted, within the PLA matrix. In Figure 9a, a comparison of tensile strength shows that the 95 PLA/5 α-Cel composite exhibits a 31% decrease in strength relative to pure PLA. However, when 5% and 10% of α-Cel grafted with LAC or ε-CL are incorporated, the tensile strength increases by 48% and 31% for LAC-grafted composites, and by 29% and 2% for ε-CL-grafted composites, respectively. When the α-Cel content is increased to 10% without grafting, it shows a 26% improvement in tensile strength; these results show higher tensile strength than PLA. This indicates that the interfacial adhesion between cellulose and PLA is enhanced when PLA composites are blended with LAC or ε-CL and grafted onto the surface of α-Cel fibers. This results in an improvement in the tensile strength [29]. On the other hand, incorporating FBA-Cel in proportions of 5% and 10% in the PLA matrix increased the percentage difference by 50% and 44% in the tensile strength compared with the pure matrix. This behavior suggests a significant improvement in the interfacial interaction between the cellulose fiber and the polymeric matrix. Incorporating LAC-g-FBA-Cel into the polymer matrix at 5% and 10% by weight reduced the tensile strength by 82% and 97%, respectively. Similarly, adding ε-CL-grafted FBA-Cel led to a 7% and 12% decrease in tensile strength. This reduction in tensile strength may be due to several factors. One potential cause is the lack of compatibility between PLA and the grafted fibers, possibly due to poor adhesion at the interface, which compromises the stress transfer from the matrix to the fibers, thus affecting the mechanical integrity of the composite. Another factor that could influence this behavior is the content of LAC or ε-CL in the blend. These compounds could have a dominant influence on the mechanical properties of the composite, resulting in a reduction in tensile strength due to their lower strength compared with PLA. In addition, it is important to consider the processing conditions, the grafting method, and the type of solvent used, as these factors could also significantly affect the mechanical behavior of the composite [30]. Figure 9b presents the Young’s modulus of the composite materials and pure PLA. Incorporating 5% and 10% by weight of LAC-g-α-Cel or ε-CL into the PLA matrix led to a modest increase in the Young’s modulus in some cases, while no significant variation was observed in others. This variability is due to overlapping values within the PLA error bars. However, upon analyzing the percentage changes, an increase ranging from 2% to 32% was observed with respect to neat PLA, indicating a moderate enhancement in stiffness attributed to better stress transfer at the fiber–matrix interface. Figure 9c,d presents the composite material’s maximum flexural strength and flexural modulus and the PLA matrix. Figure 9c shows the maximum stress values obtained from the graphs of the composite by adding 5% and 10% of α-Cel to the PLA matrix; the maximum stress values determined were 27.40 MPa and 38.16 MPa, respectively. This behavior suggests that the addition of α-Cel strengthens the matrix–fiber interface, improving the capacity of the composite material to resist loads. By adding the 5% and 10% LAC-g-α-Cel-grafted fibers to the neat PLA matrix, the percentage difference increase was 9% and 23%, indicating an improvement in flexural strength. However, the 5% ε-CL-g-α-Cel-grafted fiber shows the best performance with a percentage difference increase of 26%. On the other hand, by increasing the content of ε-CL-g-α-Cel to 10%, the percentage difference decreases to 4%. This suggests poor adhesion between the grafted fiber and the matrix, probably due to the grafting of ε-CL, which harms the adhesion and compatibility with the PLA matrix. When we added only 5% and 10% FBA-Cel to the composite material, the matrix showed good bending values close to 60 MPa. This suggests that the mechanical properties are improved. This indicates that the added fiber substantially improves the bending strength. The graft with LAC-g-FBA added to the PLA at 5% and 10% favors the bending strength considerably compared with the other samples, with values of 10.38 MPa and 8.07 MPa. This suggests a poor interaction between the PLA-grafted fiber and the matrix. The composite material grafted with ε-CL-g-FBA added at 5% and 10% in the matrix presents a moderately low flexural strength value. This is indicative of low fiber–matrix interaction.

The flexural modulus of the composite materials and the PLA matrix is presented in Figure 9d. The analysis of the results obtained from the evaluated samples will focus on the 90 PLA/10 α-Cel composite materials, with 5% and 10% by weight of FBA-Cel, LAC-g-α-Cel, FAB-Cel, and LAC-g-FBA-Cel added to the PLA matrix. These were shown to have the best flexural moduli, indicating higher stiffness. Analyzing the blend’s components, we can say that 10 α-Cel/90 PLA records the highest average modulus of 1685.74 MPa concerning PLA (1403.83 MPa). This sample presents morphological characteristics that favor its mechanical resistance. The flexural modulus of this material suggests that it has a dense and well-organized internal structure due to a good dispersion of the reinforcements within the polymeric matrix [1]. When the dispersed phases FBA, LAC-g-FBA, and LAC-g-α-Cel were added at 5% and 10% by weight in the matrix, a notable enhancement in the modulus was observed compared with the PLA matrix. This addition facilitates effective interaction with the matrix and promotes a uniform distribution of stresses throughout the composite material. These results suggest that the combination of PLA and the biopolymer grafted with LAC to the α-Cel and FBA-Cel fiber has contributed to having a rigid structure. These results indicate a more stable matrix that distributes stresses better, thus increasing the material’s ability to withstand loads.

**Figure 10:**
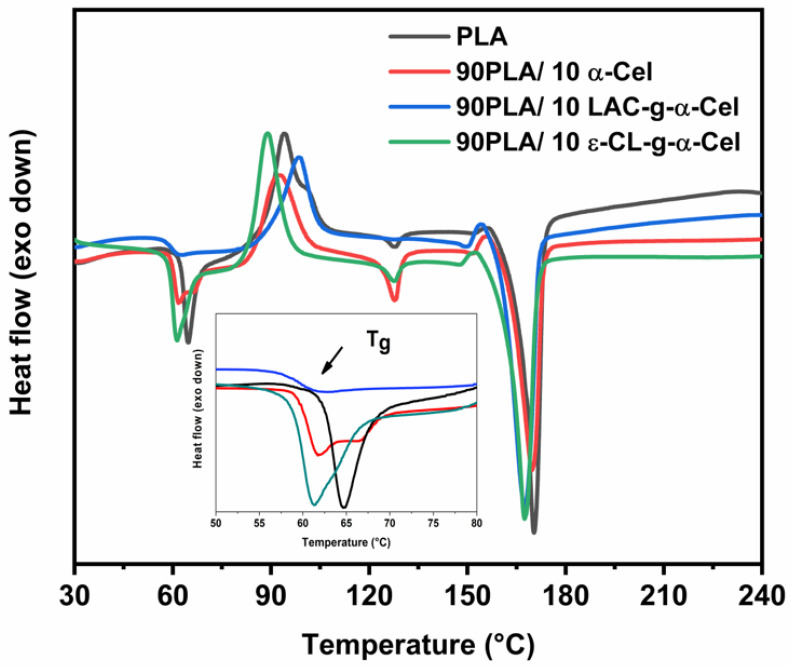
DSC curves of PLA and composite materials. In the figure insert, the T_g_ area is enlarged.

Figure 10 presents a selection of DSC thermograms for PLA and the composite material with 10% α-Cel and LAC-g-α-Cel and ε-CL-g-α-Cel. The incorporation of both grafted and ungrafted α-Cel into the PLA matrix produced a significant change in the glass transition of PLA, which decreased remarkably (around 60 °C). This behavior suggests that grafted and ungrafted fibers act as plasticizers in the PLA matrix. A double-melting band phenomenon is observed in the PLA and composite material thermograms. This behavior is well-known for PLA and is related to modifications in the crystal growth of the polymer. This band is also present in the composites and could be related to smaller crystals or a fraction of the polymer that has crystallized in a less stable form. This band indicates that the material has different crystalline domains and thermal stabilities.

Table 1 presents the glass transition (T_g_), melting (T_m_), and cold crystallization (T_c_) temperatures, as well as the melting (Δ*H_m_*) and crystallization (Δ*H_c_*) enthalpies for pure PLA and the composites reinforced with α-Cel, FBA-Cel, and grafted fibers with LAC-g-α-cellulose, ε-CL-g-α-Cel, LAC-g-FBA-Cel, and ε-CL-g-FBA-Cel. The data reveal that pure PLA exhibits a T_g_ of 63.28 °C. However, the incorporation of α-Cel, FBA-Cel, and the grafted fibers in proportions of 5% and 10% by weight resulted in a slight decrease in the T_g_, indicating better compatibility between the components, which permits increased freedom of movement for the polymer chains at the interface between the matrix and the reinforcement [8]. Regarding the cold crystallization temperature (T_c_), pure PLA shows a value of 94 °C. In contrast, the samples containing 95 PLA/5 α-Cel and 90 PLA/10 α-Cel and composite materials with 5% and 10% FBA-Cel grafted with LAC and ε-CL show a reduction in T_c_. This trend suggests that incorporating these additives improves the interfacial adhesion between the grafted fibers and the PLA matrix, which in turn reduces both the T_g_ and T_c_, which agrees with the findings reported previously [31]. However, in PLA composites with 5% and 10% α-Cel grafted with LAC or ε-CL, as well as in composites with 5% and 10% FBA, a significant increase in the crystallization temperature (T_c_) is observed, reaching values of 117.8 °C and 111.0 °C, respectively. This suggests good compatibility due to the uniform distribution of the fibers. The modified fibers improve the nucleation and interaction with PLA, which accelerates the crystallization process and requires less cooling for the material to start crystallizing. Regarding the enthalpy of crystallization (Δ*H_c_*), PLA composites with 5% and 10% CL-g-FBA-Cel show an increase in Δ*H_c_* compared with pure PLA, suggesting a higher induction of crystallinity due to the presence of ε-CL in the fibers. On the other hand, the melting temperature (T_m_) of pure PLA is 170 °C, and the inclusion of α-Cel, FBA, and grafted fibers does not significantly alter this value. However, the enthalpy of fusion (Δ*H_m_*) tends to decrease in most composite materials, indicating lower crystallinity. This could be due to the formation of less ordered crystals or a lower amount of crystals [32]. The crystallinity percentages in the composites show a decrease, suggesting that the addition of α-Cel, FBA-Cel, and grafted fibers affects the crystalline packing of PLA. This structural alteration may increase the ductility of the composites. However, the incorporation of LAC-g-FBA-Cel as a grafting agent induces a significant increase in the crystallinity of the PLA matrix, particularly at concentrations of 5% and 10%. This effect can be attributed to the ability of lactic acid to act as a nucleation promoter, facilitating the growth of crystalline regions and thus improving the propensity of the material to crystallize.

**Figure 11:**
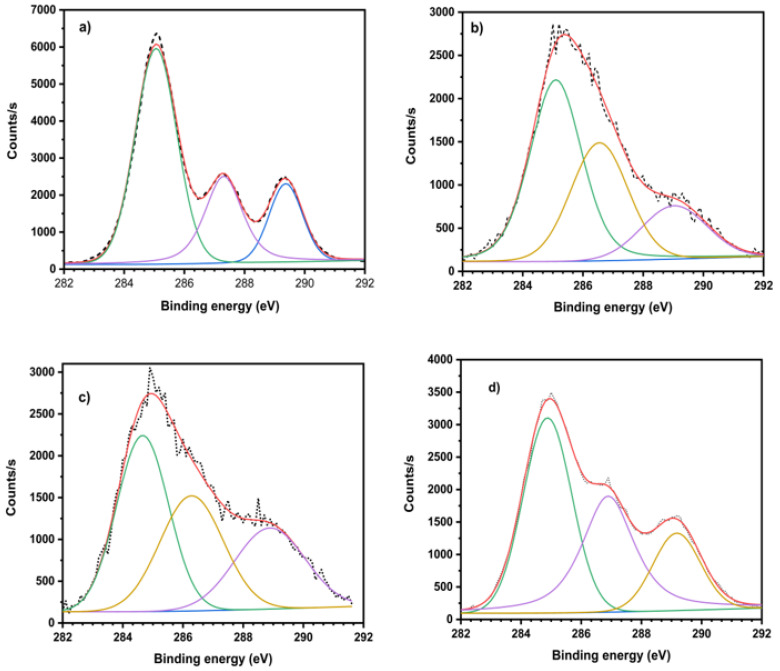
XPS of neat PLA, composite materials, and the decomposition of the C1s band of (**a**) PLA, (**b**) 90 PLA/10 α-Cel, (**c**) 90 PLA/10 LAC-g-α-Cel, and (**d**) 90 PLA/10 ε-CL-g-α-Cel.

Figure 11 shows the XPS studies of PLA, α-Cel, LAC-g-α-Cel, and ε-CL-g-α-Cel-grafted materials. The spectra have the main band at approximately 285 eV, corresponding to the carbon components. Table 2 presents the data obtained by X-ray photoelectron spectroscopy (XPS) applied to samples composed of PLA, α-Cel, and LAC-g-α-Cel and ε-CL-g-α-Cel-grafted materials. The analysis allows us to identify the functional groups present on the surface of the materials, the atomic percentage of the elements involved, and the oxygen-to-carbon (O/C) ratio in each sample. In the case of pure PLA, the bands corresponding to carbon (C1S) are observed at binding energies of 285.05, 287.30, and 289.37 eV, which are associated with different carbon bond states, such as C-C, C-O, and C=O. The oxygen bond energies (O1S) are recorded at 532.12 and 533.69 eV, characteristic of PLA chains. The O/C ratio in this sample is 0.58, a relatively low value, due to the PLA structure, which mainly contains oxygen in the form of esters (-C=O). For the 90 PLA/90 α-Cel sample, which consists of a PLA matrix with non-grafted cellulose, the binding energies of C1S remain at 285.05, 287.30, and 289.37 eV, while those of oxygen O1S are located at 532.02 and 533.03 eV. Incorporating non-grafted cellulose slightly increases the O/C ratio to 0.62, showing cellulose’s oxygen-rich structure and the hydroxyl groups’ presence. In the 90 PLA/90 LAC-g-α-Cel sample, where PLA has been grafted with lactic acid, a significant increase in the O/C ratio is observed, reaching 0.82. This increase suggests a higher number of oxygenated groups, such as esters and hydroxyls, confirming the successful grafting of lactic acid onto the cellulose surface. Finally, for the 90 PLA/90 ε-CL-g-α-Cel sample, where PLA has been grafted with ε-CL, an increase in the O/C ratio is also observed, reaching 0.68, although in a lower proportion than in the case of lactic acid. This is due to the incorporation of oxygenated groups through ε-CL but in a lower amount than grafting with lactic acid. The data obtained by XPS confirm that grafting lactic acid and ε-CL into the PLA matrix increases the number of oxygenated groups on the surface, which can improve the interaction of these materials with other components. This can enhance these materials’ interaction with other elements, making them suitable for applications in composite materials and biomaterials.

## 4. Conclusions

In this work, the morphology of PLA composites with grafted α-Cel fibers shows good adhesion between the fiber and the matrix, with no evidence of interfacial separation. In contrast, the composites with both grafted and ungrafted FBA-Cel exhibit interfacial fractures and voids, indicating weak adhesion between the fibers and the PLA matrix. Fourier transform infrared spectroscopy verified the fibers’ grafting, where the carbonyl group (C=O) band from 1750 cm⁻^1^ to 1732 cm⁻^1^ confirmed the formation of ester groups on the α-Cel fibers. Additionally, the decrease in intensity of the band corresponding to the hydroxyl group (OH) around 3342 cm⁻^1^ confirms the formation of ester bonds on the fiber surface. Regarding the mechanical properties of the study performed, the incorporation of 5% and 10% of α-Cel grafted with LAC or ε-CL increased the tensile strength by 48% and 31% for the LAC-grafted composites and by 29% and 2% for the ε-CL-grafted composites, respectively, compared with pure PLA. Regarding the thermal properties, differential scanning calorimetry analysis showed that incorporating α-Cel, FBA-Cel, and grafted fibers reduces the thermal properties of the composites, improving the compatibility with the PLA matrix.

## Figures and Tables

**Table 1:** Summary of the DSC data of PLA, α-Cel, FBA-Cel, and the composite material.

Sample	T_g_ (°C)	T_c_ (°C)	Δ*H_c_* (W/g)	T_m_ (°C)	Δ*H_m_* (W/g)	X_c_ (%)
PLA	63.28	94.65	21.19	170.43	44.95	25.54
95 PLA/5 α-Cel	62.14	93.40	28.43	169.4	40.25	13.37
90 PLA/10 α-Cel	59.32	91.97	20.02	169.63	32.05	14.37
95 PLA/ 5 LAC-g-α-Cel	59.19	100.45	32.49	167.87	41.50	10.19
90 PLA/ 10 LAC-g-α-Cel	57.64	97.43	15.19	166.31	35.50	24.26
95 PLA/ 5 ε-CL-g-α-Cel	58.82	98.71	38.78	164.44	45.71	7.84
90 PLA/ 10 ε-CL-g-α-Cel	57.55	91.13	29.72	167.20	39.28	11.42
95 PLA/5 FBA-Cel	57.77	117.81	19.21	149.33	29.61	11.77
90 PLA/10 FBA-Cel	61.03	114.99	16.72	148.84	31.67	17.88
95 PLA/ 5 LAC-g-FBA-Cel	57.36	101.18	10.59	164.43	46.33	40.45
90 PLA/ 10 LAC-g-FBA-Cel	57.72	91.79	2.41	164.97	45.41	51.37
95 PLA/ 5 ε-CL-g-FBA-Cel	59.07	89.52	25.72	167.19	36.08	11.72
90 PLA/ 10 ε-CL-FBA-Cel	57.60	86.01	23.41	166.04	33.62	12.19

**Table 2:** Binding energy, atomic percentage, and O/C ratio of PLA and composite materials by X-ray photoelectron spectroscopy.

Sample	Binding Energy (eV)	Atomic Percentage (%)	Rate O/C
C1S	285.05	36.30	
	287.30	15.97	
	289.37	10.83	
O1S	532.12	20.37	
	533.69	16.53	0.58
C1S	285.09	31.07	
	286.55	20.47	
	289.03	10.26	
O1S	532.02	21.45	
	533.03	16.75	0.62
C1S	285.0	22.63	
	286.28	18.04	
	288.89	14.15	
O1S	532.19	39.18	
	533.88	6.00	0.82
C1S	284.88	26.54	
	286.90	22.47	
	289..17	10.51	
O1S	531.89	11.75	
	533.43	28.73	0.68

## Data Availability

The original contributions presented in the study are included in the article, further inquiries can be directed to the corresponding author.

## References

[1] Auras R., Lim L.T., Selke S.E.M., Tsuji H. (2011). Poly (Lactic Acid): Synthesis, Structures, Properties, Processing, and Applications.

[2] Zhou C., Shi Q., Guo W., Terrel L., Qureshi A., Hayes D., Wu Q. (2013). Electrospun bio-nanocomposite scaffolds for bone tissue engineering by cellulose nanocrystals reinforcing maleic anhydride grafted PLA. ACS Appl. Mater. Interfaces.

[3] Chen L., Qiu X., Deng M., Hong Z., Luo R., Chen X., Jing X. (2005). The starch grafted poly (L-lactide) and the physical properties of its blending composites. Polymer.

[4] Zhou L., Ke K., Yang M.B., Yang W. (2021). Recent progress on chemical modification of cellulose for high mechanical-performance Poly (lactic acid)/Cellulose composite: A review. Compos. Commun..

[5] Teramoto Y., Yoshioka M., Shiraishi N., Nishio Y. (2002). Plasticization of cellulose diacetate by graft copolymerization of ε-caprolactone and lactic acid. J. Appl. Polym. Sci..

[6] Lönnberg H., Zhou Q., Brumer H., Teeri T.T., Malmström E., Hult A. (2006). Grafting of cellulose fibers with poly (ε-caprolactone) and poly (l-lactic acid) via ring-opening polymerization. Biomacromolecules.

[7] Lönnberg H., Fogelström L., Zhou Q., Hult A., Berglund L., Malmström E. (2011). Investigation of the graft length impact on the interfacial toughness in a cellulose/poly (ε-caprolactone) bilayer laminate. Compos. Sci. Technol..

[8] Muiruri J.K., Liu S., Teo W.S., Kong J., He C. (2017). Highly biodegradable and harsh polylactic acid–cellulose nanocrystal composite. ACS Sustain. Chem. Eng..

[9] Gupta A., Katiyar V. (2017). Cellulose functionalized high molecular weight stereocomplex polylactic acid biocomposite films with improved gas barrier, thermomechanical properties. ACS Sustain. Chem. Engin..

[10] Yu Y., Gao X., Jiang Z., Zhang W., Ma J., Liu X., Zhang L. (2018). Homogeneous grafting of cellulose with polycaprolactone using quaternary ammonium salt systems and its application for ultraviolet-shielding composite films. RSC Adv..

[11] Canché-Escamilla G., los Santos-Hernández D., Andrade-Canto S., Gómez-Cruz R. (2005). Obtención de celulosa a partir de los desechos agrícolas del banano. Inf. Tecnológica.

[12] Chen L., Xie Z., Zhuang X., Chen X., Jing X. (2008). Controlled release of urea encapsulated by starch-g-poly (L-lactide). Carbohydr. Polym..

[13] Kowalski A., Duda A., Penczek S. (2000). Kinetics and mechanism of cyclic esters polymerization initiated with tin (II) octoate. 3. Polymerization of L, L-dilactide. Macromolecules.

[14] Carlsson L. (2014). Surface Modification of Cellulose by Covalent Grafting and Physical Adsorption. Doctoral Dissertation.

[15] (2022). Standard Test Method for Tensile Properties of Plastics.

[16] (2017). Standard Test Methods for Flexural Properties of Unreinforced and Reinforced Plastics and Electrical Insulating Materials.

[17] Wang G., Zhang D., Li B., Wan G., Zhao G., Zhang A. (2019). Strong and thermal-resistance glass fiber-reinforced polylactic acid (PLA) composites enabled by heat treatment. Int. J. Biol. Macromol..

[18] Benahmed A., Azzaoui K., El Idrissi A., Belkheir H., Said Hassane S.O., Touzani R., Rhazi L. (2022). Cellulose acetate-g-polycaprolactone copolymerization using diisocyanate intermediates and the effect of polymer chain length on surface, thermal, and antibacterial properties. Molecules.

[19] Thakur V.K., Thakur M.K., Gupta R.K. (2013). Development of functionalized cellulosic biopolymers by graft copolymerization. Int. J. Biol. Macromol..

[20] Jacob J., Linson N., Mayelil-Sam R., Maria H.J., Pothan L.A., Thomas S., Kabdrakhmanova S., Laroze D. (2024). Poly (lactic acid)/nanocellulose biocomposites for sustainable food packing. Cellulose.

[21] Hosen M.S., Rahaman M.H., Gafur M.A., Ahme A.N. (2017). Development of Thermal Properties and Surface Morphology of Poly (lactic acid)/Chitosan blend with Microcrystalline Cellulose obtained from natural Jute Fiber. Int. Res. J. Pure Appl. Chem..

[22] Hospodarova V., Singovszka E., Stevulova N. (2018). Characterization of cellulosic fibers by FTIR spectroscopy for their further implementation to building materials. Am. J. Anal. Chem..

[23] Lafia-Araga R.A., Sabo R., Nabinejad O., Matuana L., Stark N. (2021). Influence of lactic acid surface modification of cellulose nanofibrils on the properties of cellulose nanofibril films and cellulose nanofibril–poly (Lactic acid) composites. Biomolecules.

[24] VP S., Mohanty S., Nayak S.K. (2016). Effect of poly (lactic acid)-graft-glycidyl methacrylate as a compatibilizer on properties of poly (lactic acid)/banana fiber biocomposites. Polym. Adv. Technol..

[25] Abdelrazek E.M., Hezma A.M., El-Khodary A., Elzayat A.M. (2016). Spectroscopic studies and thermal properties of PCL/PMMA biopolymer blend. Egypt. J. Basic Appl. Sci..

[26] Huda M.S., Mohanty A.K., Drzal L.T., Schut E., Misra M. (2005). “Green” composites from recycled cellulose and poly (lactic acid): Physico-mechanical and morphological properties evaluation. J. Mater. Sci..

[27] Khoo R.Z., Chow W.S. (2017). Mechanical and thermal properties of poly (lactic acid)/sugarcane bagasse fiber green composites. J. Thermoplast. Compos. Mater..

[28] Hou A.L., Qu J.P. (2019). Super-Toughened Poly(Lactic Acid) with Poly(ε-caprolactone) and Ethylene-Methyl Acrylate-Glycidyl Methacrylate by Reactive Melt Blending. Polymers.

[29] Awal A., Rana M., Sain M. (2015). Thermorheological and mechanical properties of cellulose reinforced PLA bio-composites. Mech. Mater..

[30] Negaresh M., Javadi A., Garmabi H. (2024). Poly (lactic acid)/poly (ε-caprolactone) blends the effect of nanocalcium carbonate and glycidyl methacrylate on interfacial characteristics. Front. Mater..

[31] Zhang C., Salick M.R., Cordie T.M., Ellingham T., Dan Y., Turng L.S. (2015). Incorporation of poly (ethylene glycol) grafted cellulose nanocrystals in poly (lactic acid) electrospun nanocomposite fibers as potential scaffolds for bone tissue engineering. Mater. Sci. Eng. C.

[32] Goffin A.L., Raquez J.M., Duquesne E., Siqueira G., Habibi Y., Dufresne A., Dubois P. (2011). From interfacial ring-opening polymerization to melt processing of cellulose nanowhisker-filled polylactide-based nanocomposites. Biomacromolecules.

